# Virtual Screening and Biomolecular Interactions of CviR-Based Quorum Sensing Inhibitors Against *Chromobacterium violaceum*

**DOI:** 10.3389/fcimb.2018.00292

**Published:** 2018-09-04

**Authors:** Vinothkannan Ravichandran, Lin Zhong, Hailong Wang, Guangle Yu, Youming Zhang, Aiying Li

**Affiliations:** State Key Laboratory of Microbial Technology, Shandong University–Helmholtz Institute of Biotechnology (SHIB), School of Life Science, Shandong University, Qingdao, China

**Keywords:** *Chromobacterium violaceum*, quorum sensing, quorum sensing inhibition, virtual screening, biofilm inhibition and microscale thermophoresis

## Abstract

The rise of bacterial multi drug resistance becomes a global threat to the mankind. Therefore it is essential to find out alternate strategies to fight against these “super bugs.” Quorum sensing (QS) is a cell-to-cell communication mechanism by which many bacteria regulate their biofilm and virulence factors expression to execute their pathogenesis. Hence, interfering the quorum sensing is an effective alternate strategy against various pathogens. In this study, we aimed to find out potential CviR-mediated quorum sensing inhibitors (QSIs) against *Chromobacterium violaceum*. Virtual screening from a natural products database, *in vitro* biofilm and violacein inhibition assays have been performed. Biofilm formation was investigated using confocal microscopy and gene expression studies were carried out using qRT-PCR. Further, to study the biomolecular interaction of QSIs with purified CviR Protein (a LuxR homologue), microscale thermophoresis (MST) analysis was performed. Results suggested that phytochemicals SPL, BN1, BN2, and C7X have potential GScore when compared to cognate ligand and reduced the biofilm formation and violacein production significantly. Especially, 100 μM of BN1 drastically reduced the biofilm formation about 82.61%. qRT-PCR studies revealed that *cviI, cviR, vioB, vioC, vioD* genes were significantly down regulated by QSIs. MST analysis confirmed the molecular interactions between QSIs and purified CviR protein which cohere with the docking results. Interestingly, we found that BN2 has better interaction with CviR (*K*_d_ = 45.07 ±1.90 nm). Overall results suggested that QSIs can potentially interact with CviR and inhibit the QS in a dose dependent manner. Since, LuxR homologs present in more than 100 bacterial species, these QSIs may be developed as broad spectrum anti-infective drugs in future.

## Introduction

Antibiotic resistance has become a global health issue and considered to be a leading health challenge in recent years (Ferri et al., 2017). Hence, efforts have to be taken to identify novel strategies which could curb bacterial pathogenesis in order to tackle multi drug resistant (MDR) “super bugs” (Wagner et al., 2016). Bacteria coordinates their behavior through quorum sensing (QS), a mechanism that helps bacterial populations to enable harmonious responses including biofilm formation and virulence factors expressions. Since, QS regulates the virulence arsenal of many pathogenic bacteria, it seems to be a captivating drug target to combat bacterial infections (Rasmussen and Givskov, 2006; Williams, 2017). Drugs targeting the virulence pathways could curb the bacterial pathogenesis and thereby prevents the disease development.

N-acylhomoserine lactones (AHLs) and peptides are the autoinducers in gram-negative bacteria and gram-positive bacteria respectively. Furthermore, autoinducer-2 (AI-2) are reported as interspecies communication signal (Miller and Bassler, 2001). AHLs contains a homoserine lactone ring with varying length of acyl chains (C4 to C18) via amide bonds (Bassler, 2002). In LuxI/LuxR- based QS systems, AHLs are synthesized by LuxI synthases and LuxR encodes the receptor proteins. Once synthesized, AHLs will be internalized, accumulated and recognized by LuxR-type receptor and this will modulate the regulation of target genes (Paul et al., 2017).

*Chromobacterium violaceum*, a gram-negative, facultative anaerobic, non-sporing coccobacillus has a quorum-sensing system consists of CviI/CviR, a LuxI/LuxR homolog (McClean et al., 1997; Stauff and Bassler, 2011). It is demonstrated that inhibitors able to interact with CviR could prevent the nematode from *C. violaceum*-mediated killing. Hence, it is apparent that the quorum sensing plays a vital role in *C. violaceum* pathogenesis and it is established that QSIs could be potent drug candidates in the battle against MDR pathogens including *C. violaceum* (Swem et al., 2009; Chen et al., 2011).

The advantages of QSIs over conventional antibiotics are as follows. Firstly, it is believed that the pathogens would not develop resistance to QSIs as this strategy may create only no or little selective pressure to the bacteria (Defoirdt et al., 2010). Secondly, QS seems to be essential for spreading bacterial resistance as it is directly or indirectly influencing the horizontal gene transfer. Thirdly, the LuxI/LuxR homologs have been reported in more than 100 Gram-negative bacterial species and over 200 different Gram-negative bacteria have been described to use AHLs as QS signals. Thereby QSIs may have the competence to be a broad range anti-virulent drugs (Adonizio et al., 2006). Taken together, interfering this mechanism would have an astounding impact over the bacterial resistance and its control (Uroz et al., 2009; Kalia, 2015). Numerous studies have been published related to quorum sensing inhibitors (QSIs) which rationalize the capability of this strategy (Ren et al., 2005; Rasmussen and Givskov, 2006; Ni et al., 2008; Kalia, 2013; Brackman and Coenye, 2015; Coughlan et al., 2016; Delago et al., 2016).

Natural products have always been fascinating source for the drug discovery. It is reported that more than 80% of drugs were natural products or inspired by a natural compound (Harvey, 2008). It is evident that almost half of the drugs approved in last two decades are based on natural products (Butler, 2008). Hence, it is crucial to screen natural products to discover potential QSIs against MDR pathogens. Despite the fact, several studies revealed that numerous plant extracts and natural products inhibit the quorum sensing of various pathogens (Adonizio et al., 2006; Vattem et al., 2007; Bouyahya et al., 2017; Paul et al., 2017), in-depth investigations are much essential to take-up these QSIs to the next level of drug discovery.

Here, we report high-throughput virtual screening of QSIs against CviR, the quorum regulator of *C. violaceum* and their biological evaluation through *in vitro* assays including qRT-PCR. To the best of our knowledge, this is the first study to discuss the molecular interactions of QSIs with purified quorum sensing target protein, CviR using microscale thermophoresis (MST) analysis.

## Materials and methods

### Bacterial strains and growth conditions

*C. violaceum* 31532, *E.coli* BL21 (DE3) were used in this study. All the bacterial strains were grown in Luria-Bertani (LB) medium, and *C. violaceum* 31532 and *E.coli* BL21 (DE3) were grown at 30° and 37°C respectively, for 24 h. Quorum sensing inhibitors (QSIs) Sappanol (SPL), Butein (BN1), Bavachin (BN2), and Catechin 7-xyloside (C7X) were purchased from Chemfaces, China.

### High throughput virtual screening (HTVS)

The virtual screening was performed against CviR using Schrodinger software (Maestro v10.6, Glide module) to screen the natural product database containing 4687 compounds. The energy minimized 3D ligand file was prepared using LigPrep module (Friesner et al., 2006). The three-dimensional structure of CviR protein was retrieved from Protein Data Bank (PDB: 3QP1 and 3QP5). Coordinates of CviR structure was prepared by using protein preparation wizard. Docking was performed using GLIDE (Grid Based Ligand Docking with Energetics) module in Schrodinger suite. Grid files were generated using the C_6_HSL, the native ligand (C_6_HSL) to the center of both the grid boxes. Tyr 80, Trp 84, Asp 97, and Ser 155 were found to be the active site residues. The compounds were subjected to HTVS ligand docking using the pre-computed grid files and then XP docking was also performed for top ranking compounds. The XP docking helps to remove the false positives with much stricter scoring function than the HTVS. Hits having least GScore (Glide score) and more number of H-bonds were analyzed further. To investigate the binding pocket of LuxR homologs, CviR from *C. violaceum* and LasR from *Pseudomonas aeruginosa* were compared using RCSB PDB Protein Comparison Tool.

### Biofilm inhibition assay

The effect of QSIs on biofilm formation was measured by microtitre plate assay (O'toole and Kolter, 1998). Briefly, overnight cultures (0.4 OD at 600 nm) of *C.violaceum* were added into 1 mL of fresh LB medium and grown with or without QSIs with varying concentrations (1, 10 and 100 μM) for 24 h at 30°C. After incubation, microtitre plates were washed with PBS (_p_H 7.4) to remove the free-floating planktonic cells. The biofilm was stained using 200 μL of 0.1% crystal violet (CV) solution. After 15 min, CV solution was removed and 200 μL of 95% ethanol was added. The biofilm was then quantified by measuring the absorbance at OD 470 nm using microplate reader (Infinite M200, Tecan).

### Violacein quantification assay

Production of violacein pigment by *C. violaceum* in the presence and absence of QSIs was analyzed by violacein extraction and quantification (Blosser and Gray, 2000). Briefly, overnight culture (OD_600_ nm = 0.1) was incubated in conical flask containing LB broth with or without QSIs (1, 10, and 100 μM) and incubated at 30°C for 24 h. Bacterial cells were then collected and the pellet was dissolved in 1 mL DMSO. Cell debris was removed by centrifugation at 13,000 g for 10 min and the absorbance of soluble violacein was read at 585 nm using microplate reader (Infinite M200, Tecan).

### Confocal laser scanning microscopy (CLSM) studies

Confocal Laser Scanning Microscopy (CLSM) analysis of the *C.violaceum* biofilms was performed as described by (Zhao and Liu, 2010). Static biofilms were grown on a glass cover slips (1: 100 diluted culture of *C.violaceum* inoculated in LB broth and incubated overnight at 30°C in stationary condition) in 6-well cell culture plates either with or without QSIs (100 μM). The developed biofilms were washed twice to remove loosely bound cells and stained with FITC-ConA for 15 min. Cells were rinsed twice in PBS to remove the excess stains and the adhered cells were analyzed using CLSM (Zeiss L800, Japan) with the excitation and emission wavelength set at 488 and 520 nm respectively.

### qRT-PCR studies

Total RNA was extracted from *C. violaceum* biofilm cells using the RNAprep Pure Kit (Tiangen, China) as per manufacturer's instructions. Biofilm cells were grown in 1 mL LB medium with or without QSIs (100 μM) at 30°C for 24 h. Total RNA was extracted with the RNA isolation kit (TIANGEN Biotech Co., Ltd., Beijing, China). Primers for *cviI, cviR, vioB, vioC, vioD*, and *rpoD* (Table S1) were synthesized by Sangon Biotech (Shanghai, China). Total RNA was used as a template for the reverse transcription reaction using a Prime Script RT Reagent Kit (TaKaRa, Japan) at 37°C for 15 min, three times (reverse transcription), and at 85°C for 5 min (reverse transcriptase inactivation) as per the manufacturer's protocol, with a total volume of 20 μL.

The qRT-PCR was performed according to the manufacturer's protocol for the SYBR® Premix Ex TaqTM II Kit (TaKaRa, Japan). Reverse transcriptase was used as a template for RT-qPCR, and the total reaction system (20 μL) was made up as the following: 10 μL SYBR^®;^ Premix Ex TaqTM II (2×), 0.8 μL forward primer, 0.8 μL reverse primer, 0.4 μL ROX Reference Dye (50×), 2 μL DNA template, and 6 μL double-distilled H_2_O (ddH_2_O). Afterwards, qRT-PCR was performed using Applied Biosystems Quant Studio™ 3 Real-Time PCR System (Applied Biosystems Inc., CA, USA). The reaction conditions are as follows: pre-denaturation at 95°C for 30 s, followed by 40 cycles of denaturation at 95°C for 5 s and annealing and extension at 60°C for 30 s. *rpoD* was used as an internal reference. The relative mRNA expression of all the genes were calculated using the 2–ΔCt method. The experiment was independently conducted 3 times.

### Expression and purification of CviR

The DNA fragment encoding CviR (GQ398094) was amplified using the primers 5′-CGATATTATTGAGGCTCACAGAGAACAGATTGGTGGATCCATGGTGATCTCGAAACCCATCAACG-3′ and 5′-TAGCAGCCGGATCTCAGTGGTGGTGGTGGTGGTGCTCGAGTTATTCGTTCGCTACGGTCGAG-3′. Primers having homology arm were used for the Red/ET recombination (Wang et al., 2016). PCR products were cloned into the expression plasmid pET28a (Novagen) linearized with BamHI and XhoI restriction enzymes by LLHR method (linear plus linear homologs recombination) of Red/ET in GBdir as per our previous reports (Wang et al., 2016). The resulting plasmid was termed as pET28-CviR and was verified by DNA sequencing of the insert and flanking regions. Produced protein corresponds to a fusion of CviR with the SUMO tag and His-tag as well. *E. coli* BL21 (DE3) was transformed with pET28-CviR. 5L Erlenmeyer flasks containing 2L LB medium supplemented with 5 μg/ml of kanamycin were inoculated with an overnight culture of *E. coli* BL21 (DE3) pET28-CviR to an initial OD _660_ of 0.05. Growth was carried out at 37°C until the OD _660_ of 0.4. The temperature was then lowered to 18°C and growth continued until an OD_660_ of 0.6 – 0.8, and then induced with 0.1 mM IPTG and growth was continued overnight at 18°C. All subsequent manipulations were conducted at 4°C. The cells were harvested by centrifugation at 4,200 rpm and ultrasonicated for 14 min in 80 mL of washing buffer with 14% of machine power (250 mM Tris-HCl, 150 mM NaCl, 20 mM imidazole, 5% glycerol, _p_H 7.8) to break the cell wall for purification. Further, samples were centrifuged at 11,000 rpm for 1 h and washed twice with washing buffer after flow-through the supernatant through Ni-sepharose 6 Fast flow (GE Healthcare, USA. 20 mL wash buffer was used to elute the CviR protein once digested with ULP proteinase. The protein was stored at −80°C for further use.

### MST analysis

All the compounds were analyzed with the concentration gradient of 50 μM with 20 μM of CviR which was labeled Monolith NT™ Protein Labeling Kit RED–NHS (Cat Nr: L001) before instrumental analysis. LED power was 20% and MST optimized buffer was used for the analysis (50 mM Tris-HCl, 150 mM NaCl, 10 mM MgCl2, 0,05% Tween-20).

Analysis was performed on Monolith NanoTemper (NT) 115 and its accessory, i.e., standard-treated 4 μL volume glass capillaries were employed to measure the molecular interaction (NanoTemper Technologies GmbH, Munich, Germany). Means of fluorescence intensity obtained by the MST measurements were fitted and the resultant *K*_d_ values were given together with an error estimation from the fit by the built-in formula of NT 1.5.41 analysis software (Cai et al., 2017).

### Statistical analysis

Graph pad prism software (version 6.01) was used for statistical analysis. One way ANOVA and multiple comparisons were carried out wherever required. *P*-values (<0.05 and <0.01) were considered as statistically significant. All the assays were conducted in triplicates and the results were expressed as mean ± SD.

## Results

### Computational studies

#### Molecular interaction of QSIs against CviR

**Figure 1:**
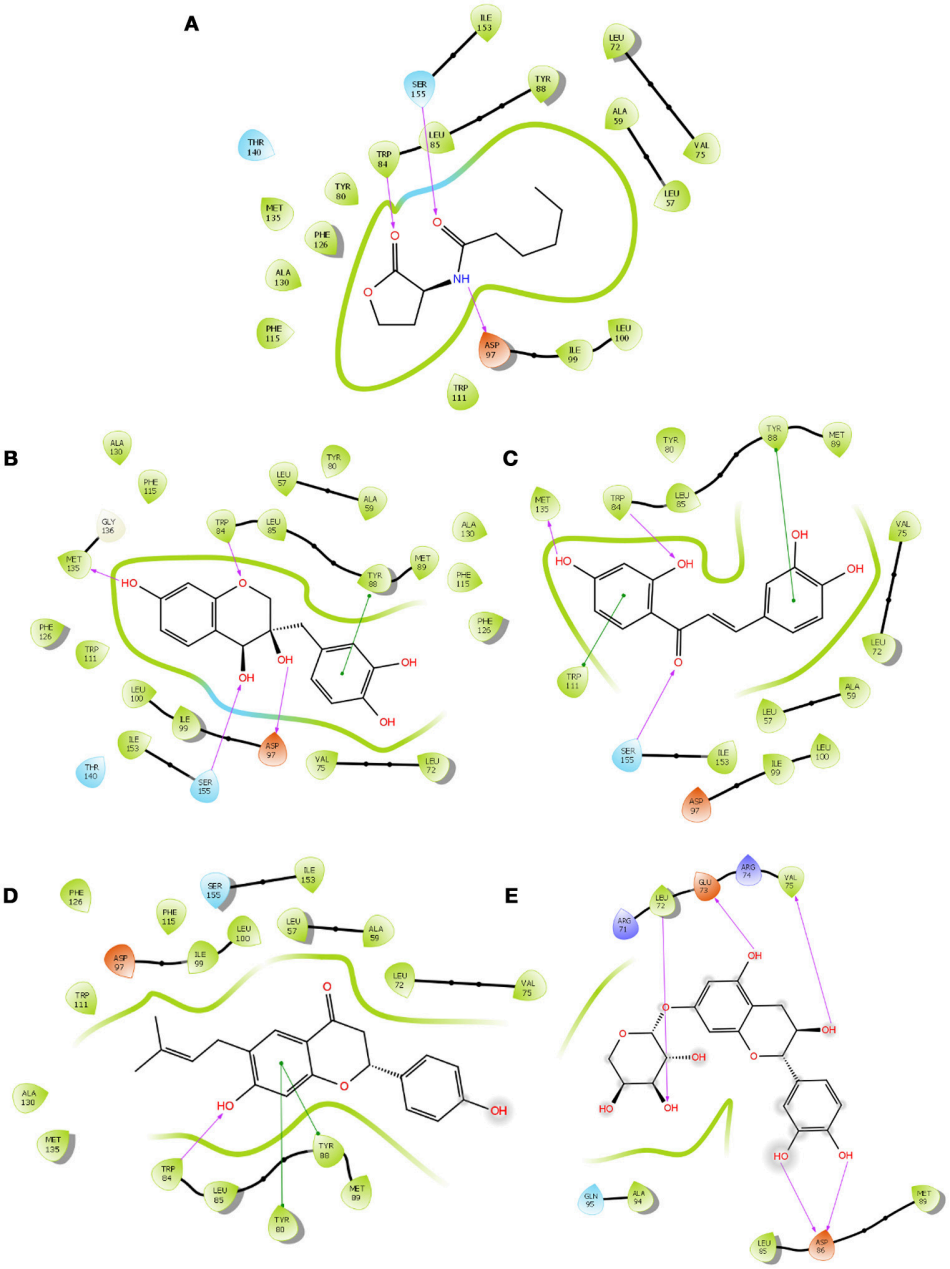
The 2D interaction diagrams of QSIs against CviR. The purple arrows shows the H-bonds whereas the green lines shows the π-π stacking. **(A)** is for interaction map of C_6_HSL, **(B)** for interaction map of SPL, **(C)** for interaction map of BN1, **(D)** for interaction map of BN2 and **(E)** for interaction map of C7X against CviR. Most of the QSIs have at least one common bond forming aminoacid with C_6_HSL (Trp 84, Asp 97, Ser 155) and all of them have least GScore than C_6_HSL.

**Figure 2:**
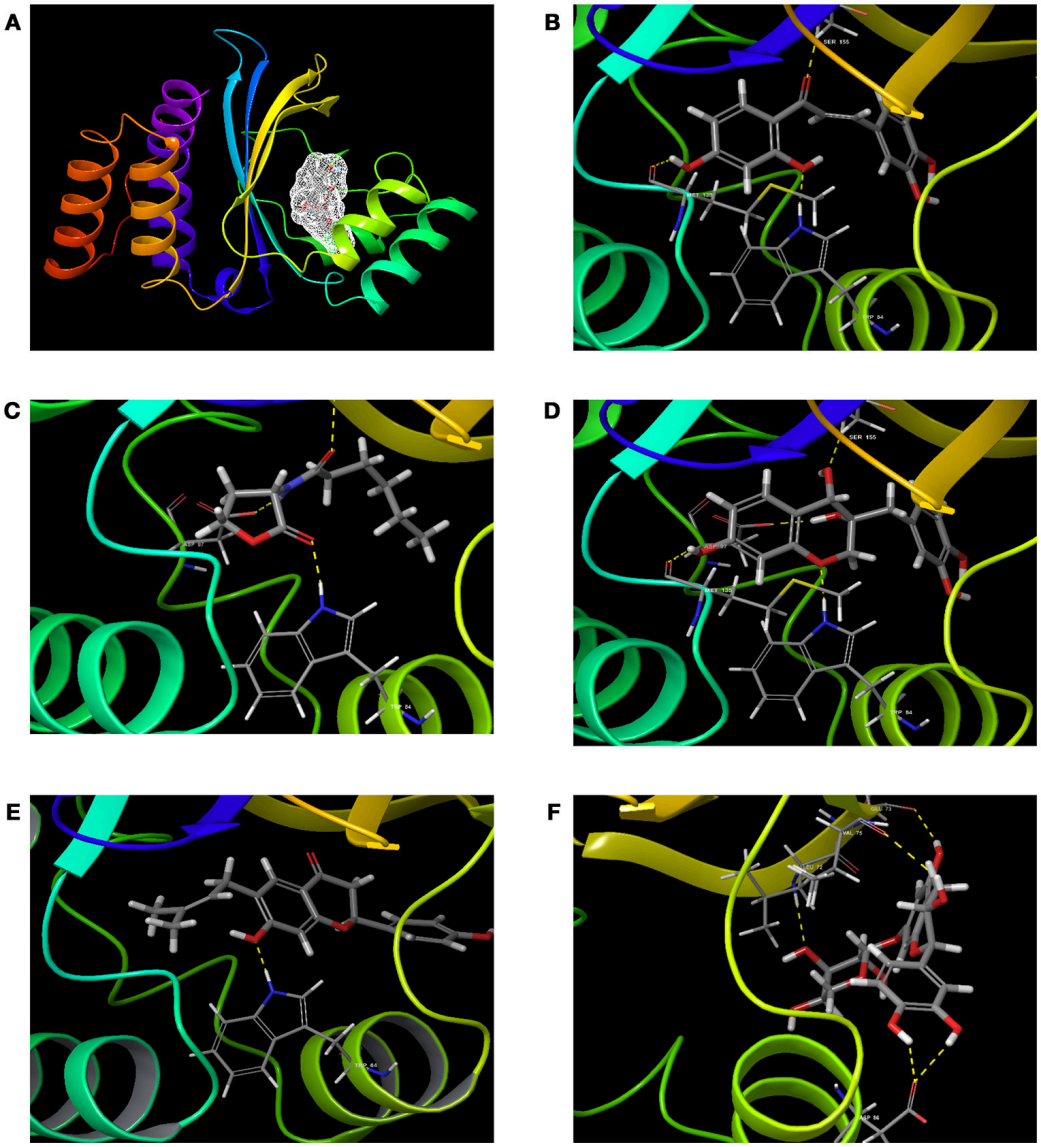
The 3D molecular interactions of QSIs against CviR **(A)** is for CviR, the LuxR homolog of *C. violaceum* where the ligand binding pocket is depicted in light gray **(B)** is for molecular interaction of C_6_HSL with CviR (3 H bonds) **(C)** for molecular interaction of SPL (4 H bonds) **(D)** for molecular interaction of BN1 (3 H bonds) **(E)** for molecular interaction of BN2 (1 H bond) and **(F)** for molecular interaction of C7X against CviR (4 H Bonds). H-bonds represented in yellow dotted line.

To screen quorum sensing inhibitors against CviR, QS regulator of *C. violaceum*, virtual screening was performed using a natural product database. GScore for the native ligand (C_6_HSL) was −7.052 and C_6_HSL was able to form three H-bonds with Trp 84, Asp 97, Ser 155 (Figures 1A–E) which was used as reference value and pattern of interaction for the pose analysis. SPL, BN1, BN2, C7X were having the GScore −12.140, −11.246, −8.056, −7.414 (Table 1) respectively. SPL was able to form 4 H-bonds with amino acids Trp 84, Asp 97, Met 135 and Ser 155 along with a pi-pi stacking with Tyr 88. BN1 was able to form 3 H-bonds with Trp 84, Met 135 and Ser 155 along with 2 pi-pi stacking with Trp 84 and Tyr 88. Whereas, BN2 was able to form only one H-bond with two pi-pi interaction with Tyr80 and Tyr 88 (Figures 2A–F). In contrast, C7X has a very unusual pattern of interaction and it was able to form 4 H-bonds with Glu73, Val 75, Asn 77, and Asp 86. After pose analysis, based on GScore and H-bond forming ability, the ligands were chosen for further studies.

**Table 1:** Docking analysis of Quorum sensing inhibitors against CivR, the quorum regulator of *Chromobacterium violaceum*.

**S.No**	**Name**	**Structure**	**GScore**	**Number of H-bonds**	**Bond forming amino acids**
1	C_6_HSL	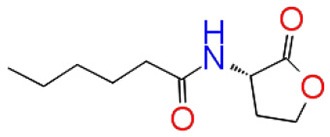	−7.052	3	Trp 84 Asp97Ser 155
2	Sappanol	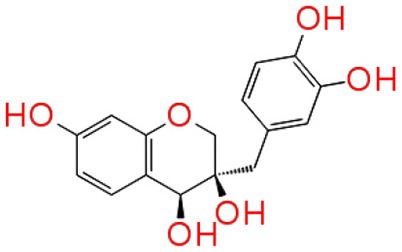	−12.140	4	Trp 84 Asp 97 Met 135 Ser 155
3	Butein	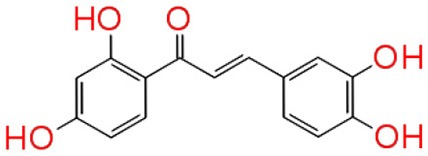	−11.246	3	Trp 84 Met 135 Ser 155
4	Bavachin	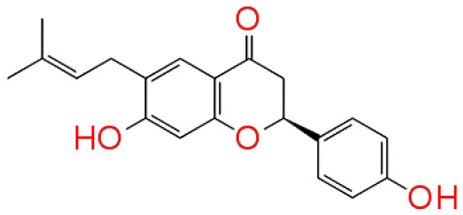	−8.056	1	Trp 84
5	Catechin 7-xyloside	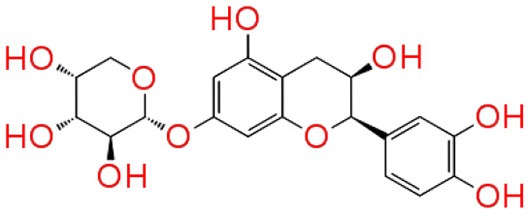	−7.414	4	Glu73 Val 75 Asn 77 Asp 86

### Comparative analysis of CviR vs. LasR

**Figure 3:**
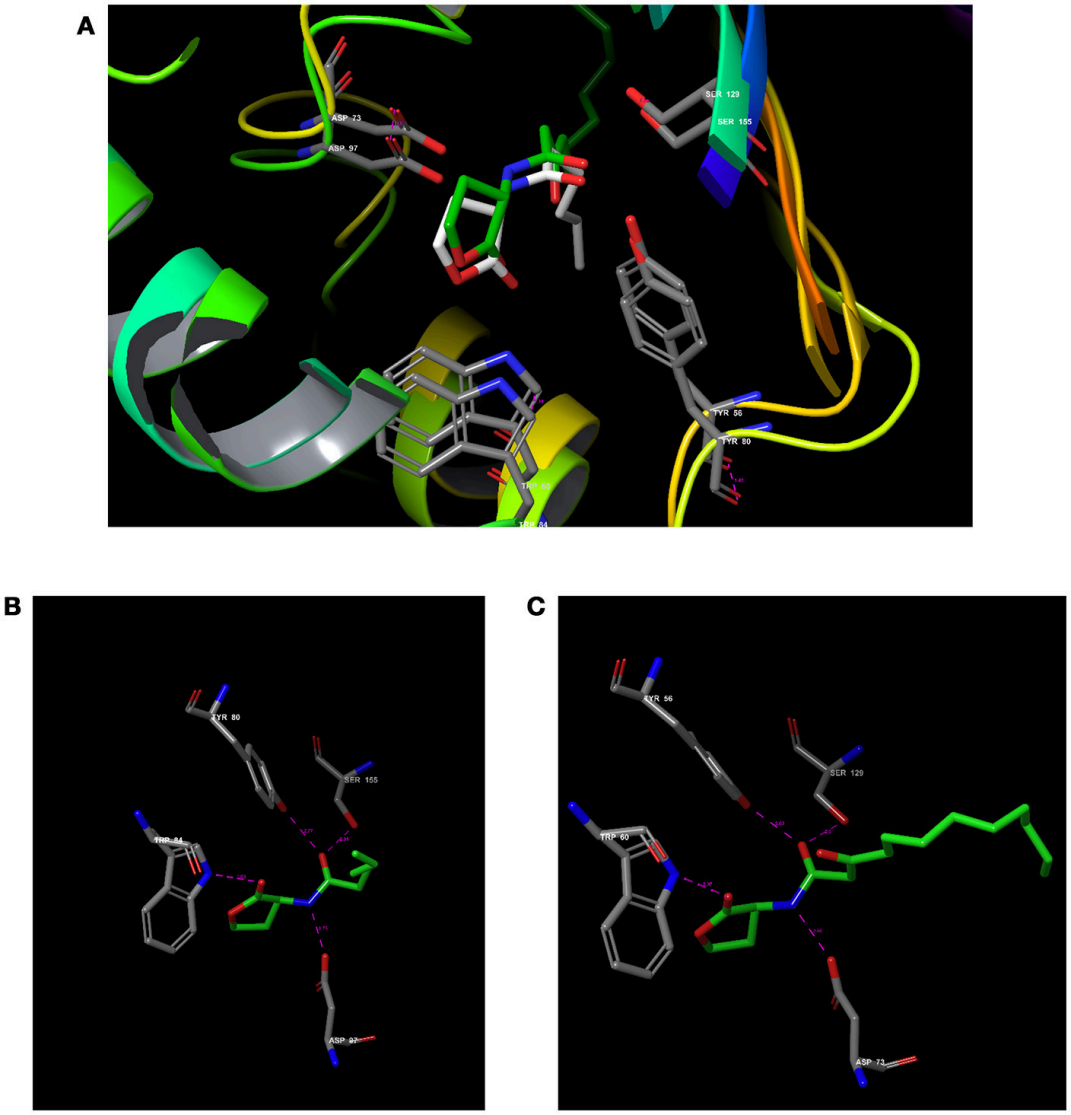
Comparative analysis of CviR with LasR using RCSB PDB Protein Comparison Tool. **(A)** Molecular comparison of CviR vs LasR. **(A)** The alignment of the active site residues (Tyr, Trp, Asp, Ser) which are crucial for the interactions. **(B)** The molecular interaction of (N-hexanoyl-L-Homoserine lactone (C_6_HSL) against CviR **(C)** The molecular interaction of N-(3-Oxododecanoyl)-L-homoserine lactone (3-Oxo-C_12_-HSL) with LasR.

To verify how close the binding pocket LuxR homologs are, the PDB structure of CviR and LasR were analyzed. As expected, both cognate ligands were interacting with the very similar amino acids in both receptors. C_6_HSL was interacting with Tyr 80, Trp 84, Asp 97, and Ser 155 in CviR and 3-oxo-C_12_HSL was interacting with Tyr 56, Trp 60, Asp 73 and Ser 129 (Figure 3A). Further, positional changes of these residues were calculated and found to have very minute change. The distance between Tyr 80-56 was 1.43 Å and the distance between Trp84-60 was 1.14 Å (Figures 3B,C). Asp 97-73 were in a distance of 1.00 Å. Surprisingly, Ser155-129 were in a distance less than 1 Å (0.75 Å). Further, we found that SPL and BN1 were able to interact with Ser 155, which is crucial for CviR and LasR as well.

### Influence of QSIs on biofilm, growth, and violacein

**Figure 4:**
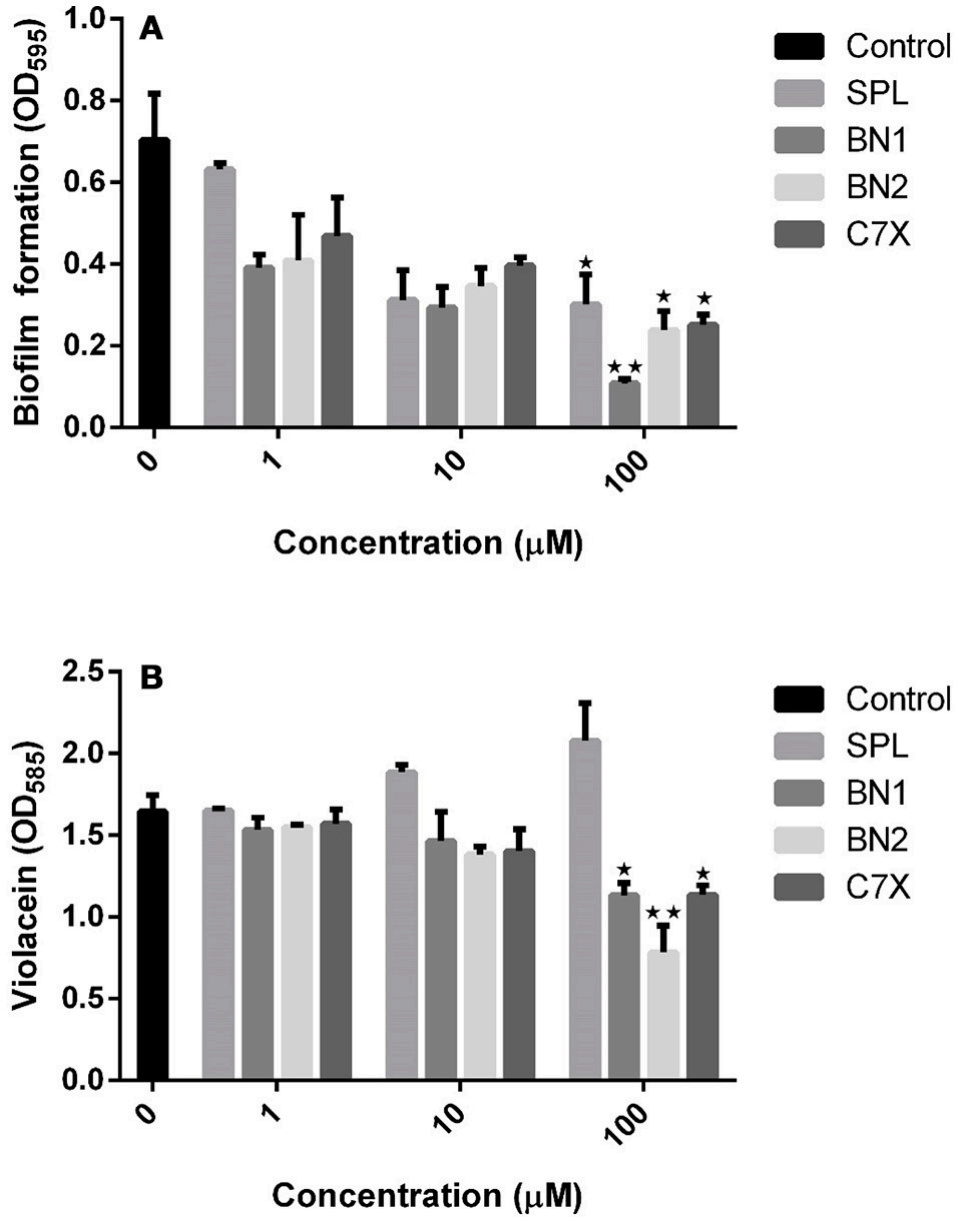
The effect of QSI on biofilm formation and violacein. **(A)** The biofilm formation was inhibited by QSIs. Especially, BN2 and C7X have reduced the biofilm formation significantly whereas BN1 decreased the biofilm formation drastically (~50%). **(B)** The similar trend was followed in violacein production also. Though all the three QSIs reduce the violacein production, the SPL found be increased. *P < 0.05 and **P < 0.01.

It is essential to verify the efficacy of QSIs against quorum sending regulated phenotypes in *C.violaceum*. Biofilm is one of the major factor that is under the control of quorum sensing and plays a crucial role in pathogenesis and drug resistance. All the tested QSIs reduced the biofilm formation significantly at varying concentrations (1, 10 and 100 μM). Except C7X, all the QSIs reduced more than 50% of biofilm at 10 μM concentrations (Figure 4A). Especially, BN1 significantly reduced the biofilm formation about 82.61% when supplied with 100 μM. Whereas, BN2 and C7X reduced the biofilm by about 66 and 64.26% respectively with the similar treatment. To differentiate the quorum sensing inhibition activity of these QSIs from antibiotic activity, the growth was analyzed. Except SPL, none of the QSIs found to have influence on the growth of *C. violaceum* (Figure S1). Violacein, a purple pigment produced by the *C. violaceum* which is reported to be under the control of QS mechanism via *vioABCDE* operon. Violacein quantification analysis revealed that all the tested QSIs have potentially suppressed the violacein production. BN2 reduced the violacein drastically by 52.50% when treated with 100 μM (Figure 4B). A concentration-dependent reduction in the violacein production was observed. Unfortunately, SPL was found to increase the production of violacein by 14.51 and 26.26% when treated with 10 and 100 μM respectively.

### Confocal studies

**Figure 5:**
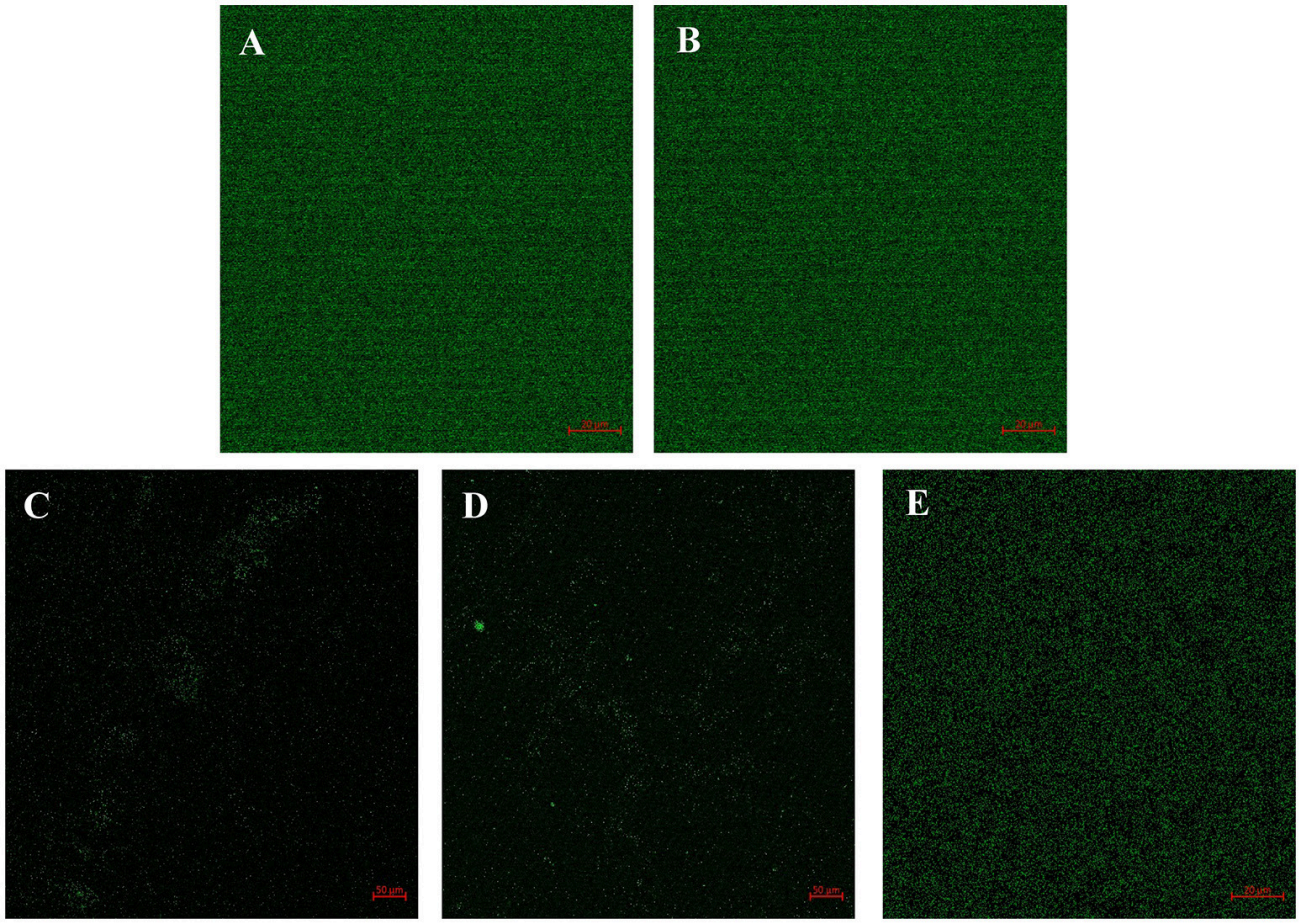
Confocal image shows the efficacy of QSIs to curb the biofilm formation. **(A)** Control **(B)** SPL **(C)** BN1 **(D)** BN2 and **(E)** C7X.

The efficacy of the QSIs on biofilm development was examined using the confocal laser scanning microscopy (CLSM). It was found that all the QSIs except SPL were negatively regulating the biofilm formation when treated with 100 μM concentrations (Figures 5A–E). To be specific, BN2 and BN2 have significantly reduced the biofilm formation when administered with 100 μM.

### Gene expression studies

**Figure 6:**
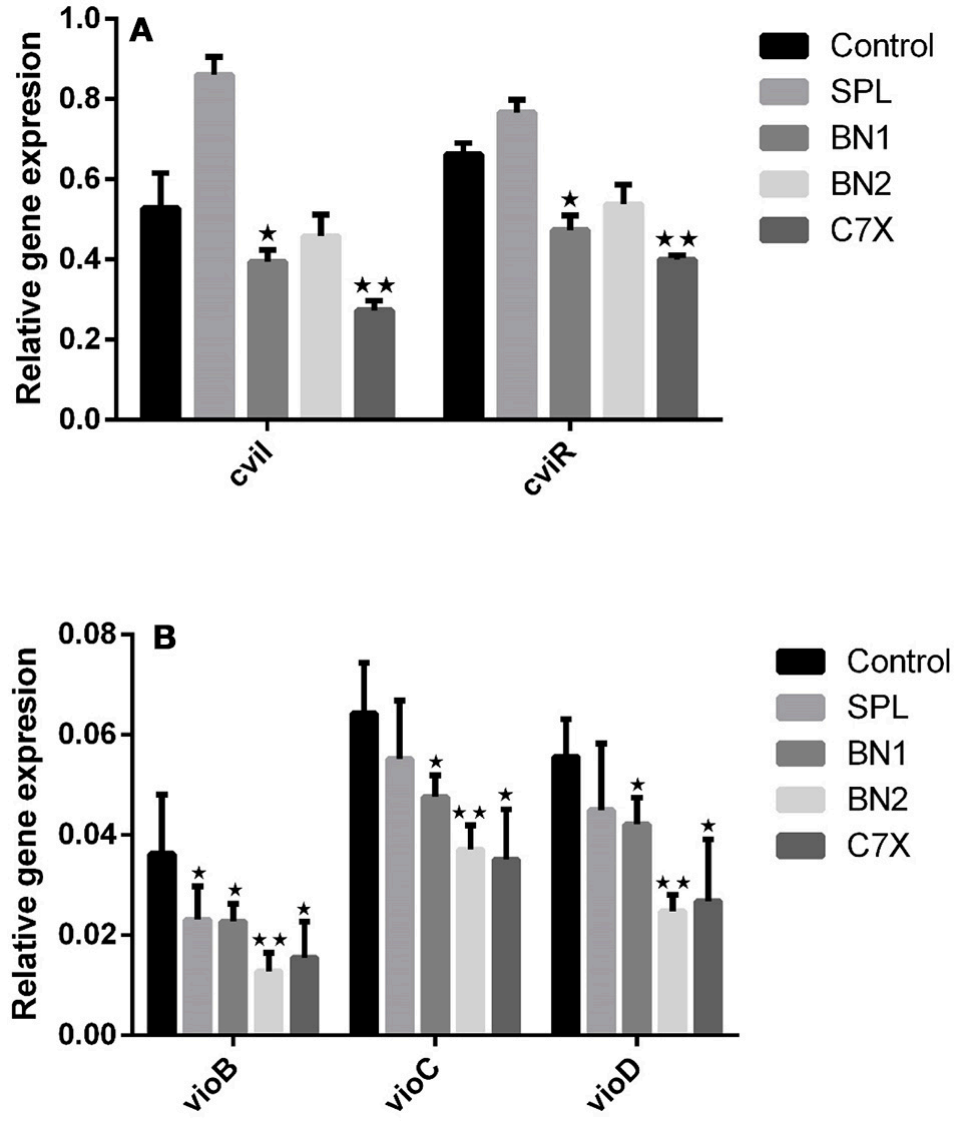
The influence of QSIs on the genes which are under the control of CviI/CviR mediated quorum sensing. **(A)** The *cviI* and *cviR* genes were significantly suppressed by BN1 and C7X. **(B)** The genes involved in violacein biosynthesis were significantly suppressed by BN2. *P < 0.05 and **P < 0.01.

To investigate the impact of QSIs (100 μM) on the genes expression related to *C.violaceum* quorum sensing, qRT-PCR studies have been performed. First of all, the effect of QSIs on *cviI* and *cviR* was evaluated. Data suggest that the BN1 and C7X were able to significantly suppress the expression of *cviI* (Figure 6A). Whereas in case of cviR, the similar pattern of decrement was observed which is comparable to that of *cviI*. It is noteworthy, that SPL increased the expression of *cviI* but comparatively less in *cviR*.

To study further the effect of QSIs on *vioABCD* operon, genes including *vioB, vioC, vioD* were analyzed. All the QSIs were significantly reduced the expression of *vioB, vioC, vioD* genes (Figure 6B). Especially, BN2 decreased the expression of these genes very significantly. Surprisingly, SPL also decreased the expressions.

### Expression and purification of CviR

**Figure 7:**
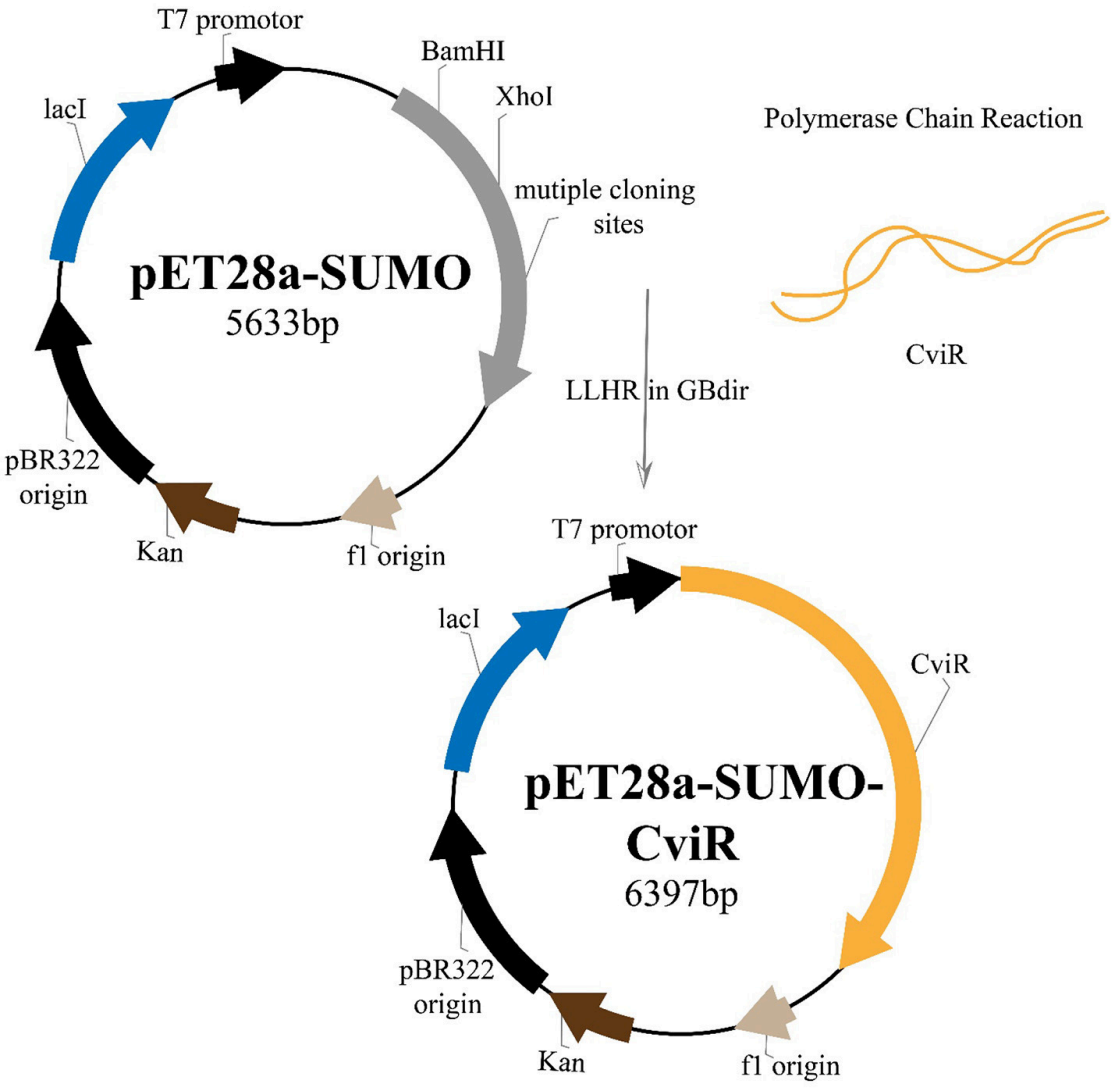
Plasmid construction and the recombineering strategy followed for the heterologous expression of CviR protein.

RecET based direct cloning and Redαβ based recombineering were used for heterologous expression of CviR Protein. The *cviR* gene was cloned into the expression vector pET28a and the protein was expressed in *E. coli* (Figure 7). The purification were carried out under different conditions of cell growth and buffers. The purified protein was further investigated by polyacrylamide gel electrophoresis (PAGE) (Figure S2).

### Microscale thermophoresis

**Figure 8:**
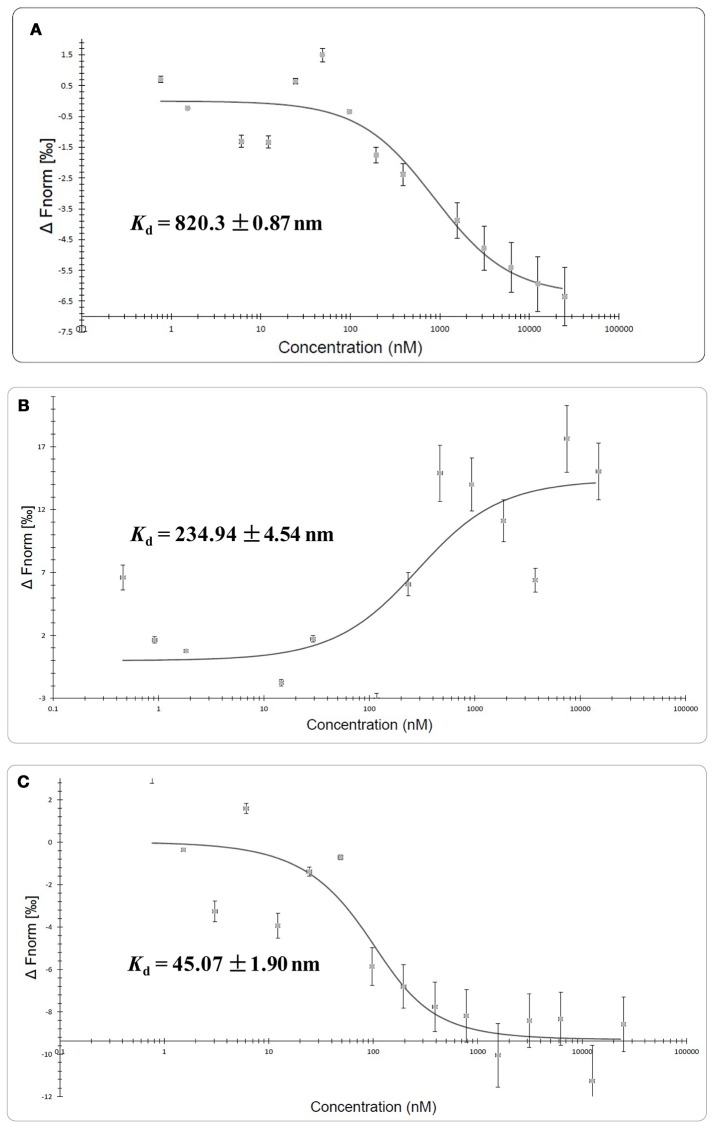
Molecular interaction of QSIs using microscale thermophoresis analysis. **(A)** Molecular interaction of SPL **(B)** molecular interaction BN1 **(C)** molecular interaction BN2. Unfortunately we could not find any such interaction with C7X.

MST experiment was carried out to detect the molecular interaction between QSIs and CviR. Differences in normalized fluorescence of the bound and unbound state will allow determination of the fraction bound and thus the dissociation constant is calculated. All values are multiplied by a factor of 1,000 which yields the relative fluorescence change in per thousand. MST results suggested that all the QSIs except C7X have significant binding ability. It is noteworthy to mention that the dissociation constant (*K*_d_) of BN2 is 45.07 ±1.90 nm (Figure 8C). Surprisingly, the F_norm_ value of BN1 is increased when the concentration increased and having a sigmoidal curve suggests a competitive interaction pattern (Figure 8B).

## Discussion

Due to misuse and overuse of antibiotics along with complex bacterial drug resistance mechanisms, antimicrobial resistance has emerged as a global threat (Soukarieh et al., 2018). According to WHO's Global Antimicrobial Surveillance System (GLASS), occurrence of MDR infections was found among half a million people all around the world. Resistance to penicillin has raised up to 51% whereas ciprofloxacin resistance raised from 8 to 65%. The recent report from GLASS confirms that there is a serious situation of antibiotic resistance worldwide (Tornimbene et al., 2018). Many case reports on *C.violaceum* infections were published with variety of health complications and it is resistant to a broad range of antibiotics including rifampin, vancomycin, ampicilin and cephalosporins (Fantinatti-Garboggini et al., 2004; Justo and Durán, 2017). Hence, alternatives to antibiotics is the “need of the hour” which will ultimately reduce morbidity, mortality and economic burden (Laxminarayan et al., 2016). Recently, disarming the bacterial virulence seems to be a potential alternate strategy to combat MDR (Rangel-Vega et al., 2015; Mookherjee et al., 2018). AHL mediated quorum sensing inhibition was reported to be effective in many pathogens including *C.violaceum, Pseudomonas aeruginosa* (Kim et al., 2015; Deryabin and Inchagova, 2018; Pérez-López et al., 2018; Soukarieh et al., 2018; Zhou et al., 2018). Since, natural products are the alluring sources of drug discovery, we intended to screen the CviR inhibitors from phytochemicals.

Virtual screening results suggested that numerous chemical moieties were able to interact with the CviR. Based on the GScore and the ability to form H-Bonds, four natural products have been chosen for further studies. Our *in silico* data revealed that molecular docking is in consistent with Crystallographic structures and in coherence with previous report (Kimyon et al., 2016). Generally, LuxR-type proteins are homodimers and each monomer consists of two domains, a ligand-binding domain (LBD) and a DNA-binding domain (DBD). Upon reception of cognate signal via LBD, they will undergo certain conformational changes, thereby allowing gene expression (Chen et al., 2011). All the QSIs SPL, BN1, BN2, C7X have better GScore than that of C_6_HSL (−7.052). SPL and BN1 have a very similar pattern of interaction alike C_6_HSL along with H-bond Met 135. BN2 has a single H-bond with Trp 84, one of the key residue in the binding pocket of C_6_HSL and it is observed to have two pi-pi interactions as well (Figures 1D, 2E). It is speculated that, BN1 and BN2 may induce a closed conformation of CviR, hence it cannot interact with the DNA and thus inhibiting the QS as similar as chlorolactone (CL). It is demonstrated that CL potentially inhibits the *C.violaceum* QS by interacting with Trp 84 and Asp 97 along with a pi-pi stacking with Tyr 88 which is very similar to the pattern of interaction of BN1 (Swem et al., 2009). Whereas C7X has entirely different pattern of interaction with 4H bonds and surprisingly, C7X has interaction with Arg 74, which was reported to be present in DBT. Hence, it is hypothesized C7X could inhibit the QS by occupying the DBD and inducing a closed conformation.

Our findings showed that the AHLs (C_6_ HSL and 3-oxo-C_12_HSL) have four crucial point of interactions such as lactone carbonyl group which forms an H-bond with Trp84 residue, the acyl group amine forms a H-bond with Asp 97 and the carbonyl oxygen which forms H-bonds with Tyr80 and Ser155 which coheres with the results of Ahmed et al. (2013) (Figures 3A–C). Further, it is found that Ser155_CviR_ and Ser129_LasR_ were in a distance less than 1 Å (0.75 Å) and this suggested that Ser in the LBD must be a very essential point of interaction. Even though docking relies on many approximations, lead optimization was often in concert with evaluations and moreover this virtual screening approach saves time, manpower and cost when compared to the traditional approaches.

In AHL mediated QSIs identification process via virtual screening, biofilm formation and violacein quantification assays are the basic and crucial steps. Since violacein pigment is under the control of QS mechanism, *C.violacum* is considered to be one of the best and easily accessible biomonitor strains to screen QSIs of any origin. Our *in vitro* studies demonstrated that the QSIs have a potential influence on QS regulated phenotypes at the tested concentrations (1, 10, 100 μM) without affecting the growth (Figures 4A,B and Figure S1). Numerous studies have been reported that the plant-based natural products, such as Vanillin, Naringin, Naringenin, Quercetin, Ellagic acid and Curcumin, reduced the biofilm formation without affecting the growth (Bouyahya et al., 2017). Curcumin was reported to reduce the biofilm and virulence related traits in various uropathogens in a concentration dependent manner (Packiavathy et al., 2014). Carvacrol significantly reduced the biofilm formation (0.1–0.3 mM) of *C.violaceum* and other pathogens (Burt et al., 2014). Quercetin and quercetin-3-O-arabinoside inhibited violacein production in *C. violaceum*, at 50 and 100 μg/mL, respectively (Vasavi et al., 2014). Isoprenyl caffeate, from *manuka propolis* found to reduce violacein in agar diffusion assays (Gemiarto et al., 2015). Studies revealed that tannin rich fractions of *Terminalia catappa* inhibited violacein production (50%) at 62.5 μg per mL without significantly affecting growth (Taganna et al., 2011). It is observed that our QSIs, have suppressed the QS at minimal concentrations when compared to most of the earlier reports. To demonstrate the effect of QSIs on biofilm, CLSM studies have been performed. Results revealed that the QSIs, BN1 and BN2 have drastically reduced the biofilm (Figures 5D,E), which is in consistent with *in vitro* biofilm assay. Unfortunately, SPL increased the biofilm formation which is comparable to the control. Though many reports available on biofilm and violacein inhibition of various plant extracts in search of QSIs, the active principle responsible for such effects have not been investigated further in most of the cases. Many synthetic chemicals have also been explored for QSI activity but still they are not taken for further studies.

To investigate the efficacy of QSIs on the genes which are under the control of QS mechanism, qRT-PCR experiment was conducted. The *cviI* gene which produces the C_6_HSL and the gene *cviR* which produces CviR protein were taken into consideration. Data suggest that significant suppression by BN1 and C7X is in consistent with the *in vitro* study results. Our results are in agreement with (Burt et al., 2014) which was observed that 0.3 mM of carvacrol inhibited the *cviI* gene expression. Hence, these results indicated that QSIs inhibited the production of AHL at gene expression level.

In violacein biosynthetic pathway, the *vioB* produces a polyketide synthase, which is very essential for biosynthesis of violacein, as it catalyzes the condensation of two tryptophan derivatives. Whereas *vioD, vioC* are nucleotide-dependent monooxygenases. *vioD* seems to catalyze the hydroxylation of one tryptophan moiety, whereas *VioA* seems to catalyze an oxidative deamination in the second tryptophan moiety, and *vioC* catalyzes intermediate violacein oxidation (August et al., 2000). To study the effect of these QSIs on genes involved in violacein biosynthetic pathway, we have tested the gene expression of *vioB, vioC*, and *vioD*. All the QSIs have significantly suppressed the genes tested. BN1 significantly reduced the expression of *vioB, vioC*, and *vioD* (Figures 6A,B). It is documented that *Manuka propolis* PF5 treatment (300 μg/ml) down-regulated *vioD*, and the key residue was found to be isoprenyl caffeate (Gemiarto et al., 2015). Gene expression study showed the efficacy of QSIs in down regulating the QS related genes which play roles in biofilm formation and virulence directly or indirectly.

Further, to study the molecular interactions between these QSIs and CviR, CviR protein was expressed, isolated and purified for microscale thermophoresis (MST) analysis. For the CviR protein expression, RecET from Rac prophage mediated linear–linear homologous recombination (LLHR) method was followed as per our previous report, which can be used to clone large DNA regions directly from genomic DNA into expression vectors (Wang et al., 2016). MST is a powerful technique to measure biomolecular interactions which based on thermophoresis, the movement of molecules in a temperature gradient. This technique was reported to be highly sensitive that allows precise quantification of molecular interactions (Jerabek-Willemsen et al., 2014). MST results suggest that all the QSIs have potential molecular interaction with purified CviR (Figures 8A–C). The dissociation constant (*K*_d_) of the BN2 is 45.07 ±1.90 nm (Figure 8C). These data suggest that BNI having a very similar interaction pattern to that of C_6_HSL along with 2 pi-pi interactions, shows very significant interaction with CviR. According to Seidel et al. (2013), the fitting curve may be either S-shaper or mirror S-shaped. The reversal sign of MST amplitude (change in normalized fluorescence) depends on the chemistry of the compound that is titrated (e.g., Charge), its binding site and the conformational change induced upon binding. SPL and BN2 have negative slope suggesting interaction that don't alter the conformation significantly. Whereas the BN1 shows a positive slope suggesting a strong conformational change induced upon complex formation. Probably two pi-pi interactions play a major role in conformational change. Though C7X was not able to fit into the CviR binding pocket, we speculate that it might interfere the QS mechanism by negatively influencing the conformational changes required for the QS activation by interacting with the region near DNA binding domain (DBD) of CviR.

Overall results suggest that except SPL, BN1, BN2, and C7X significantly suppressed the QS of *C. violaceum*. Sappanol (SPL) is a 3, 4-dihydroxyhomoisoflavan, can be found in *Caesalpinia sappan*. Butein (BN1) is a chalcone, can be found in *Toxicodendron vernicifluum*. Bavachin (BN2) is a flavonoid, can be found in *Psoralea corylifolia*. Catechin-7-Xyloside (C7X) is flavan-3-ols, can be found in *Spiraea hypericifolia L*. All the natural products have their own biological activity profile. Virtual screening, *in vitro* studies, CLSM analysis of biofilm, qRT-PCR studies and molecular interaction studies using MST, suggest that BN1, BN2 significantly inhibited the CviR-mediated QS, whereas C7X might have a different mode of action and has to be explored further. Though QSIs are potential alternative to antibiotics in the battle against MDR pathogens, it is essential to have an eye on the chances for QSIs getting resistance (Kalia et al., 2014; García-Contreras, 2016).

## Conclusion

To summarize, our present data from virtual screening, docking analysis, qRT-PCR and MST measurement proved that the phytochemicals BN1, BN2, C7X inhibit CviR-mediated quorum against *C.violaceum* and represent potential CviR-mediated quorum sensing inhibitors against *C.violaceum*. Since LuxR homologs are present in more than 100 gram negative pathogens, these QSIs may be developed as a broad spectrum anti-infective drug candidates. Considering the emergence of multi drug resistant pathogens, it is very essential to develop novel drug discovery strategies to find potent drugs against these deadly pathogens. Since natural products always play a major role in medicine and human health, virtual screening of natural products against the molecular drug targets will be a productive approach. It is evident that starting with biological evaluation, gene expression studies and molecular interactions using MST, will help us to get an in-depth understanding of the mode of action of these moieties. It is concluded that BN1 and BN2 inhibiting the *C.violaceum* by interacting with LBD of CviR. In contrast, C7X interacting with DBD of CviR and show comparatively less inhibition than BN1 and BN2. Finally thus, this approach will help us to find out effective QSI against various pathogens.

## Author contributions

YZ and VR conceived the idea and planned the experiments. VR, LZ and GY performed the experiment. YZ, VR, HW and AL contributed in data interpretation, AL, YZ and VR wrote the manuscript.

### Conflict of interest statement

The authors declare that the research was conducted in the absence of any commercial or financial relationships that could be construed as a potential conflict of interest. The reviewer YGT and handling Editor declared their shared affiliation.
