# Supplementary material for: Virtual Screening and Biomolecular Interactions of CviR-Based Quorum Sensing Inhibitors Against Chromobacterium violaceum
Source: Front Cell Infect Microbiol. 2018 Sep 4;8:292. doi: 10.3389/fcimb.2018.00292 (PMC6132053; doi:10.3389/fcimb.2018.00292)
Supplement: Supplementary file 1 [file Data_Sheet_1.pdf]

Supplementary Material

Virtual screening and biomolecular interactions of CviR-based quorum sensing inhibitors against *Chromobacterium violaceum*

Vinothkannan Ravichandran, Lin Zhong, Hailong Wang, Guangle Yu, Youming Zhang *

Shandong University–Helmholtz Institute of Biotechnology, State Key Laboratory of Microbial Technology, School of Life Science, Shandong University, Qingdao - 266237, P.R. China

* **Correspondence:**
Corresponding Author: Youming Zhang
zhangyouming@sdu.edu.cn

Table S1: The primers used in this study

Gene	Primers
cviR-5RT	GGTATTGGGACGCCTGAACA
cviR-3RT	CTGGGAGTACTGGTTGAGCC
cviI-5RT	GAAACCGTCCTCGCATAAGG
cviI-3RT	CTGAAACTAAGCTGCGACAGTTG
vioB-5RT	ATGCGAATGCTCGTAATGCG
vioB-3RT	TCCTCAACGAGCTGACCAAC
vioC-5RT	TTGCTATGCATGTAGCGGGTG
vioC-3RT	CAAGGCCTTTCCCGAGTTCAC
vioD-5RT	GGAACACCTTGGCGACGTAT
vioD-3RT	GGACACGATGAGCACCTTCA
rpoD-cvir-5RT	GAATTCGGAGTCCACCGAGG
rpoD-cvir-3RT	GCATCCAGGTATGCGAGGAA

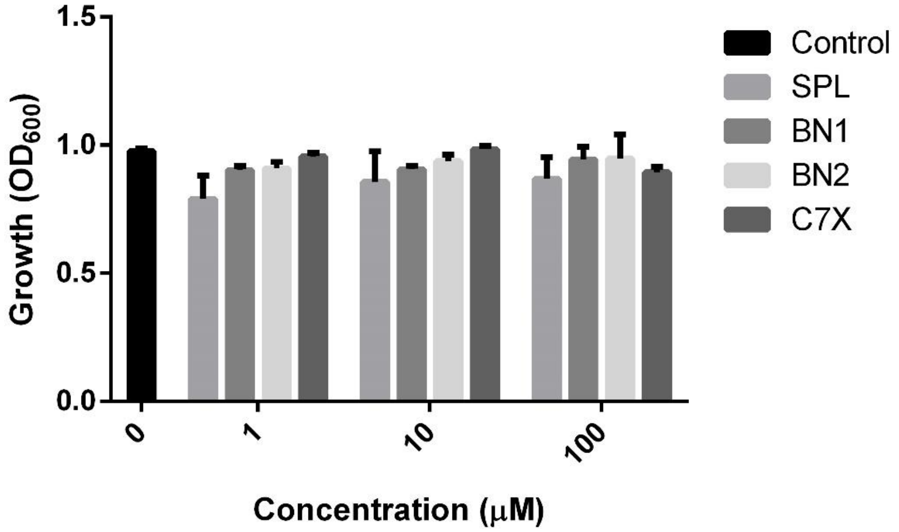

Figure S1: The influence of QSIs on the growth of *C.violaceum*. Though there is a fluctuation, there is no significant growth inhibition was observed.

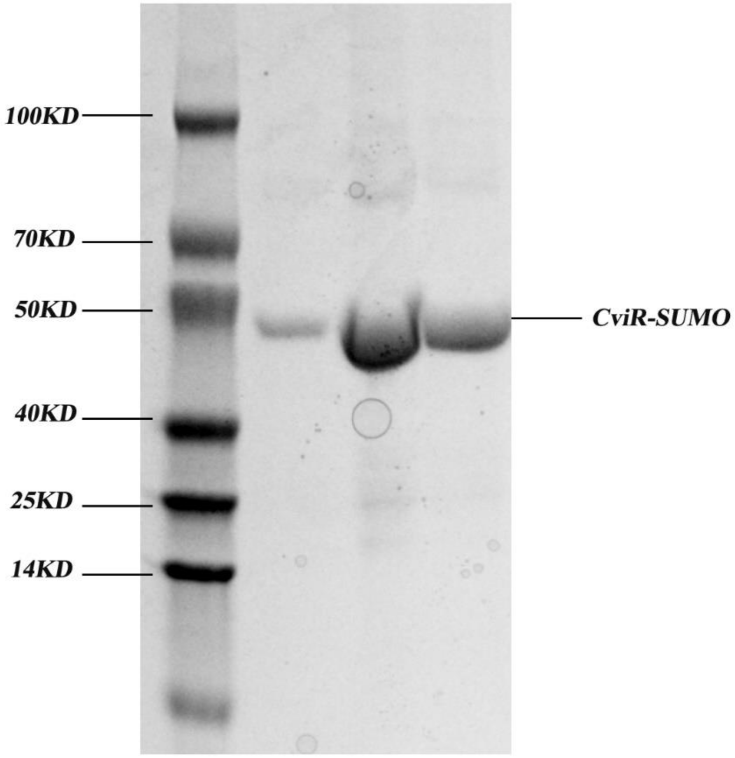

Figure S2: The purified CviR protein with SUMO.
